# *In silico* discovery of selenocysteine-containing variants of Group 4 [NiFe] hydrogenases

**DOI:** 10.3389/fmicb.2026.1925269

**Published:** 2026-09-03

**Authors:** Masamitsu Takano, Masao Inoue, Riku Aono, Anna Ochi, Hisaaki Mihara

**Affiliations:** College of Life Sciences, Ritsumeikan University, Kusatsu, Shiga, Japan

**Keywords:** bacteria, bioinformatics, hydrogenase, molecular evolution, phylogenetic analysis, selenocysteine

## Abstract

[NiFe] hydrogenases reversibly catalyze hydrogen oxidation and proton reduction at a Ni–Fe active site coordinated by four cysteine (Cys) residues in two CXXC motifs within the catalytic large subunit. A subset of these enzymes contains selenocysteine (Sec) in place of Cys in the C-terminal CXXC motif, forming [NiFeSe] hydrogenases with enhanced oxygen tolerance and hydrogen production activity. However, such Sec-containing variants have been reported only in Groups 1 and 3 [NiFe] hydrogenases. Here, we present a comprehensive dataset of Group 4 [NiFeSe] hydrogenases based on a genomic survey combined with UGA stop-codon read-through analysis. The dataset comprises 47 non-redundant protein sequences from nine bacterial phyla. Sec substitutions were exclusively identified in the N-terminal CXXC motif, including 27 CXXU-, 4 UXXC-, and 16 UXXU-type sequences, indicating the emergence of non-canonical Sec-containing motifs in Group 4. Phylogenetic analysis revealed a sporadic distribution across three distinct lineages, indicating at least three independent origins of Sec-containing variants from Cys-type ancestors. Such substitutions were found across diverse ecosystems. Genomic context analysis further suggests that these Sec-containing enzymes form energy-converting complexes, similar to those formed by other Group 4 enzymes. The Sec-containing variants co-occur with Sec biosynthesis genes and a conserved guanine residue in the apical loop of Sec insertion sequence elements. These findings provide new insights into the evolution of Sec utilization in [NiFe] hydrogenases.

## Introduction

Hydrogen metabolism is one of the most ancient and widespread biological processes (Vignais and Billoud, 2007; Peters et al., 2015; Helmbrecht et al., 2025). Hydrogenases catalyze the reversible interconversion between molecular hydrogen and protons (H_2_ ⇆ 2H^+^ + 2e^−^) and are widely distributed in microbes, functioning in respiration, fermentation, and redox homeostasis (Vignais and Billoud, 2007; Peters et al., 2015; Schuchmann et al., 2018). They are also promising biocatalysts for industrial-scale H_2_ production, a low-impact energy source (Lee et al., 2010; Kim and Kim, 2011). Among them, [NiFe] hydrogenases, characterized by a Ni–Fe catalytic center, constitute the most abundant and functionally versatile class (Fontecilla-Camps et al., 2007; Vignais and Billoud, 2007).

Structurally, [NiFe] hydrogenases share a conserved heterodimeric architecture in which the large subunit contains a Ni–Fe catalytic center and the small subunit harbors multiple iron-sulfur clusters for electron transfer (Volbeda et al., 1995; Fontecilla-Camps et al., 2007; Lubitz et al., 2014). The active site consists of a Ni atom coordinated by four cysteine (Cys) residues and is bridged to an Fe atom bearing one CO and two CN^−^ ligands, whose assembly requires specialized maturation factors (Jacobi et al., 1992; Volbeda et al., 1995). These four Cys residues are organized into two highly conserved sequence motifs, typically denoted as CXXC motifs, located in the N-terminal and C-terminal regions of the large subunit.

[NiFe] hydrogenases are phylogenetically classified into four major groups with distinct physiological functions, including uptake (Group 1), sensing (Group 2), bidirectional (Group 3), and energy-converting (Group 4) hydrogenases (Vignais and Billoud, 2007). Within this diversity, Group 4 [NiFe] hydrogenases represent a functionally distinct class of multisubunit, membrane-associated complexes that couple hydrogen metabolism to ion translocation and energy conservation (Yu et al., 2018; Schoelmerich and Müller, 2019). Group 4 [NiFe] hydrogenases also function as components of respiratory enzyme complexes, enabling efficient redox coupling. For example, in the formate hydrogenlyase complex, a [NiFe] hydrogenase is coupled with formate dehydrogenase (Fdh) to catalyze the conversion of formate to H_2_ and CO_2_ (McDowall et al., 2014; Steinhilper et al., 2022). Large-scale comparative genomic studies have revealed extensive diversity within Group 4 [NiFe] hydrogenases, which are consequently classified into subgroups 4a−4i in the HydDB scheme (Søndergaard et al., 2016).

Another notable variation within the [NiFe] hydrogenase family is the occurrence of selenocysteine (Sec)-containing enzymes, referred to as [NiFeSe] hydrogenases. To our knowledge, Sec-containing variants have been reported only in Groups 1 and 3 (Baltazar et al., 2011). In these enzymes, Sec substitutions occur predominantly within the conserved C-terminal CXXC motif of the large subunit, replacing the Cys residue that directly coordinates the Ni atom in this motif, resulting in a UXXC motif (Garcin et al., 1999; Baltazar et al., 2011; Marques et al., 2017). Sec plays a critical role in enzyme maturation, catalytic efficiency, and resistance to oxidative damage (Marques et al., 2017). In particular, Sec modulates catalytic bias to enhance proton reduction relative to H_2_ oxidation (Parkin et al., 2008).

Because Sec is encoded by recoding the UGA stop codon, its incorporation into proteins requires a specialized translational mechanism (Zinoni et al., 1987; Leinfelder et al., 1988; Forchhammer et al., 1989; Zinoni et al., 1990; Zhang et al., 2026). This process depends on a dedicated set of molecular components, including the Sec-specific tRNA (SelC), the elongation factor (SelB), a *cis*-acting RNA structure termed the Sec insertion sequence (SECIS) element, and Sec biosynthesis enzymes (SelA and SelD). Sec utilization is highly dynamic across evolution, with repeated gains and losses of selenoproteins across lineages (Zhang et al., 2006). Notably, Sec residues frequently arise through replacement of redox-active Cys residues, indicating a directional bias toward Cys-to-Sec substitution.

Given the extensive diversity of [NiFe] hydrogenases and the dynamic nature of Sec utilization, it is intriguing that Sec-containing variants appear to be restricted to specific groups and positions. In this study, we performed a comprehensive bioinformatics analysis to identify Sec-containing variants of Group 4 [NiFe] hydrogenases, including those potentially overlooked due to misannotation arising from UGA stop-codon interpretation. The resulting dataset provides detailed insights into potential Sec utilization within Group 4 [NiFe] hydrogenases and reveals that Sec-type variants occur in a rare but diverse range of bacterial genomes. Sec residues in Group 4 were found exclusively in the N-terminal motif, distinct from those in Groups 1 and 3. These findings highlight a previously unrecognized aspect of Sec utilization in [NiFe] hydrogenases.

## Materials and methods

### Sequence retrieval of Group 4 [NiFe/NiFeSe] hydrogenases

Protein sequence retrieval for Group 4 [NiFe] hydrogenase catalytic large subunits was performed using DIAMOND BLASTp version 2.1.11 (Buchfink et al., 2021) in ultra-sensitive mode against the National Center for Biotechnology Information (NCBI) non-redundant protein sequence database (April 10, 2025; Sayers et al., 2026). The following sequences were used as queries: WP_001288122.1, WP_011012581.1, WP_012572531.1, WP_011407013.1, WP_014183752.1, WP_001102321.1, WP_013174844.1, WP_013560598.1, WP_025344446.1, WP_015940516.1, WP_201769473.1 and WP_010876037.1. Short-length (amino acid length < 200) and low-score (E-value > 0.001) hits were excluded. Sequences with deletions in the core region or mutations in the two conserved CXXC motifs (allowing one Sec, His, Glu, or Asp substitution) were then filtered out based on inspection of multiple sequence alignments generated using MAFFT version 7.520 with the E-INS-i method (Katoh and Standley, 2013). In addition, the corresponding genome assemblies were retrieved from the NCBI assembly database (December 3, 2025) by searching for protein accession numbers. Protein sequences without genomic information were excluded.

Reconstruction of Sec-containing sequences was performed as follows. Sequences terminated at the −1 position of the conserved Cys were extended by translating downstream of the corresponding TGA stop codon to the next in-frame stop codon using SeqKit version 2.10.0 (translation table 11; Shen et al., 2016). Full-length reconstruction was verified by multiple sequence alignment as described above. Additional extensions were performed when termination occurred at the −1 position of other conserved Cys. For N-terminally truncated sequences, translation was initiated from upstream start codons in the same reading frame, and full-length Sec-containing sequences were reconstructed using the procedure described above.

Classification based on HydDB (Søndergaard et al., 2016) was used to exclude sequences assigned to Groups 1, 2, and 3, resulting in 23,473 Group 4 [NiFe/NiFeSe] hydrogenase sequences. This classification was further validated by phylogenetic analysis.

### Phylogenetic analysis of Group 4 [NiFe/NiFeSe] hydrogenases

Amino acid sequences of Group 4 [NiFe] hydrogenase catalytic large subunits were clustered at 80% sequence identity using DIAMOND cluster version 2.1.11 (Buchfink et al., 2026), resulting in 2,022 clusters. The centroid sequences were aligned using MAFFT with the E-INS-i method. Ambiguously aligned sites were subsequently trimmed using trimAl version 1.4.1 with the automated1 option (Capella-Gutiérrez et al., 2009). As outgroups, previously characterized Group 1 and Group 3 [NiFeSe] hydrogenases from *Desulfovibrio vulgaris* (recently reclassified as *Nitratidesulfovibrio vulgaris*; WP_010939204.1) and *Methanococcus voltae* (WP_013180376.1), respectively, were included. The trimmed alignment used for phylogenetic analysis contained 244 amino acid sites. A maximum-likelihood tree was constructed using IQ-TREE version 3.0.1 (Wong et al., 2026) under the Q.PFAM+I+R10 model selected by ModelFinder (Kalyaanamoorthy et al., 2017). Branch support was assessed using SH-aLRT (Guindon et al., 2010) and ultrafast bootstrap (Hoang et al., 2018) based on 1,000 replicates. The tree was visualized using iTOL version 7.2 (Letunic and Bork, 2024).

### Reconstruction of subgroup-specific phylogenetic trees

Amino acid sequences belonging to subclades encompassing Sec-type lineages and their Cys-type sister groups in Groups 4b, 4e, and 4g were extracted from the full sequence dataset based on the phylogenetic tree of Group 4 [NiFe] hydrogenase catalytic large subunits. These sequences were clustered at 100% sequence identity using DIAMOND cluster. The centroid sequences for each group were aligned using MAFFT with the E-INS-i method and subsequently trimmed using trimAl with the automated1 option. Maximum-likelihood trees were reconstructed using IQ-TREE under the following models: Group 4b, LG+R7; Group 4e, LG+I+G4; and Group 4g, LG+G4. For Groups 4b, 4e, and 4g, 163, 27, and 9 sequences were analyzed, respectively, with corresponding outgroups: Group 4a HycE from *Escherichia coli* (WP_001288122.1), Group 4e EchE from *Methanosarcina mazei* (WP_011034248.1), and Group 4c CooH from *Carboxydothermus hydrogenoformans* (WP_011344721.1).

### Genomic analysis and gene annotation

For genomic context analysis, the 15 genes upstream and downstream of the gene encoding the catalytic large subunit were annotated based on Clusters of Orthologous Groups (Galperin et al., 2025) using standalone RPS-BLAST version 2.15.0 (Wang et al., 2023) with an E-value cutoff of 10^−6^. When multiple genomes were clustered within subclades, those with more comprehensive flanking gene annotations were retained.

Genes for [NiFe] hydrogenase maturation (*hypABCDEF*) and Sec biosynthesis/incorporation (*selABD*) were identified from orthogroups inferred using OrthoFinder version 3.0.1b1 (Emms and Kelly, 2019) with the DIAMOND search mode and MAFFT/FastTree-based tree inference (Price et al., 2010).

SelC (tRNA^Sec^) and SelC^*^ (tRNA^ReC^; Vargas-Rodriguez et al., 2018) were detected using tRNAscan-SE version 2.0.12 (Chan et al., 2021). Predicted tRNAs of at least 80 nucleotides were assigned to SelC if they contained a UCA anticodon and a 3′ discriminator base of G or A, or to tRNA^ReC^ if they contained UCA or GCA anticodons and a discriminator base of U. Multiple sequence alignment with a reference tRNA^ReC^ sequence was performed using MAFFT, and highly divergent sequences were excluded.

### Taxonomy and ecosystem annotation

Taxonomic classification was performed based on the Genome Taxonomy Database (GTDB) R220 (Parks et al., 2022). Genomes not registered in GTDB were classified using GTDB-Tk version 2.4.0 (Chaumeil et al., 2022). Ecosystem information associated with metagenome-assembled genomes was manually categorized into aquatic, terrestrial, engineered, and host-associated environments based on textual metadata from NCBI BioSample records linked to each genome (Sayers et al., 2026).

### Prediction of SECIS secondary structures

mRNA sequences located downstream of the UGA codon (Sec type) or the corresponding UGC/U codon (Cys type) were manually extracted within a range of 40 to 60 nucleotides. For proteins containing the UXXU motif, mRNA sequences were extracted from the first UGA codon or the corresponding UGC/U codon. Secondary structure prediction of mRNA was performed using MXfold2 version 0.1.2 (Sato et al., 2021). Candidate SECIS elements were identified by visual inspection of SECIS-like stem-loop structures. bSECISearch (Zhang and Gladyshev, 2005) was used to support the manual predictions and to refine candidate selection when multiple SECIS-like structures were identified.

### 3D structure prediction of protein complex

The structure of the Group 4 [NiFeSe] hydrogenase complex was predicted using AlphaFold version 3.0.1 (Abramson et al., 2024). Representative amino acid sequence sets were selected as follows: Group 4b CXXU-type from Chloroflexota (MCX6010236.1–MCX6010246.1), Group 4b UXXU-type from Desulfobacterota (MDI6776830.1–MDI6776841.1), Group 4e UXXC-type from Desulfobacterota (HVO65047.1–HVO65053.1), and Group 4g CXXU-type from Thermodesulfobiota (WP_108308809.1–WP_108308817.1). For catalytic large subunits containing Sec, full-length sequences obtained after UGA read-through reconstruction were used. To avoid program errors, Sec residues were substituted with Cys residues. C-terminal regions subject to post-translational processing were removed prior to analysis. The following entries from the PDB Chemical Component Dictionary were used as ligands: NI (Ni^2+^ ion), FCO (carbon monoxide-(dicyano) iron, Fe(CN)_2_(CO)), SF4 ([4Fe−4S] cluster), FES ([2Fe−2S] cluster), FAD (flavin-adenine dinucleotide), MO (Mo atom), and MGD (molybdopterin guanosine dinucleotide). Ligand-containing JSON files were generated using alphafold3_tools version 0.1.0. Top-ranking models with horizontally oriented membrane modules were selected and visualized using PyMOL version 3.0.0 (Schrödinger).

### Statistical analysis

Enrichment was assessed using Fisher's exact test, with *p*-values calculated using the fisher.test() function in *R* version 4.4.2.

## Results

### Identification of Group 4 [NiFeSe] hydrogenases

To explore the structural and evolutionary diversity of Group 4 [NiFeSe] hydrogenases, we constructed an expanded dataset of Group 4 [NiFe/NiFeSe] hydrogenases from the NCBI genome database. A total of 23,473 non-redundant catalytic large-subunit sequences were classified into Group 4 based on phylogenetic analysis and HydDB assignment (Søndergaard et al., 2016).

**Figure 1:**
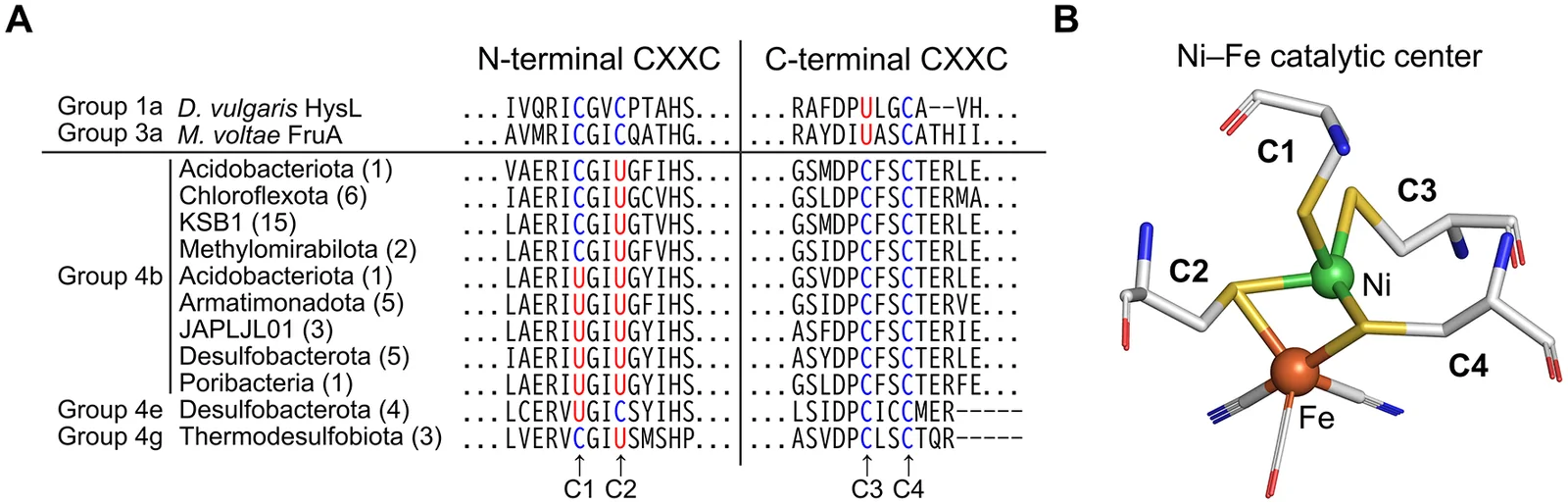
Comparison of Cys- and Sec-containing active-site motifs in the catalytic large subunits of Group 4 [NiFe/NiFeSe] hydrogenases. **(A)** Multiple sequence alignment of the N-terminal and C-terminal Ni-binding motifs. Representative sequences for Groups 1 and 3 were derived from *Desulfovibrio vulgaris* HysL (WP_010939204.1) and *Methanococcus voltae* (WP_013180376.1), respectively. For Group 4, representative sequences from each phylum and subgroup are shown. **(B)** Structure of the NiFe active-site center from a formate hydrogenlyase complex of *Escherichia coli* (PDB, 7Z0T). Ni and Fe atoms are shown as green and brown spheres, respectively. Each coordinated Cys residue is designated as C1–C4, and the corresponding sites are indicated in **(A)**.

The dataset included 47 non-redundant Sec-containing variants, of which 46 were identified via UGA stop codon read-through analysis. Notably, 44 of the 47 Sec-containing sequences were derived from metagenome-assembled genomes. Sec variants were derived from nine bacterial phyla—Acidobacteriota, Armatimonadota, Chloroflexota, Desulfobacterota, JAPLJL01, KSB1, Methylomirabilota, Poribacteria, and Thermodesulfobiota (Figure 1 and Supplementary Table 1).

Sec was consistently located in the N-terminal Ni-binding motif across all 47 sequences (Figure 1), in contrast to well-characterized Group 1 and Group 3 [NiFeSe] hydrogenases, which predominantly contain a C-terminal UXXC motif (Baltazar et al., 2011). Moreover, structural variations at the Sec-containing N-terminal motif were identified: among the 47 Sec-containing variants, 27 were classified as CXXU-type, 4 as UXXC-type, and 16 as UXXU-type (Figure 1). These results suggest that non-canonical Sec-containing motifs have emerged in Group 4 [NiFeSe] hydrogenases.

### Phylogenetic diversity of Group 4 [NiFeSe] hydrogenases

**Figure 2:**
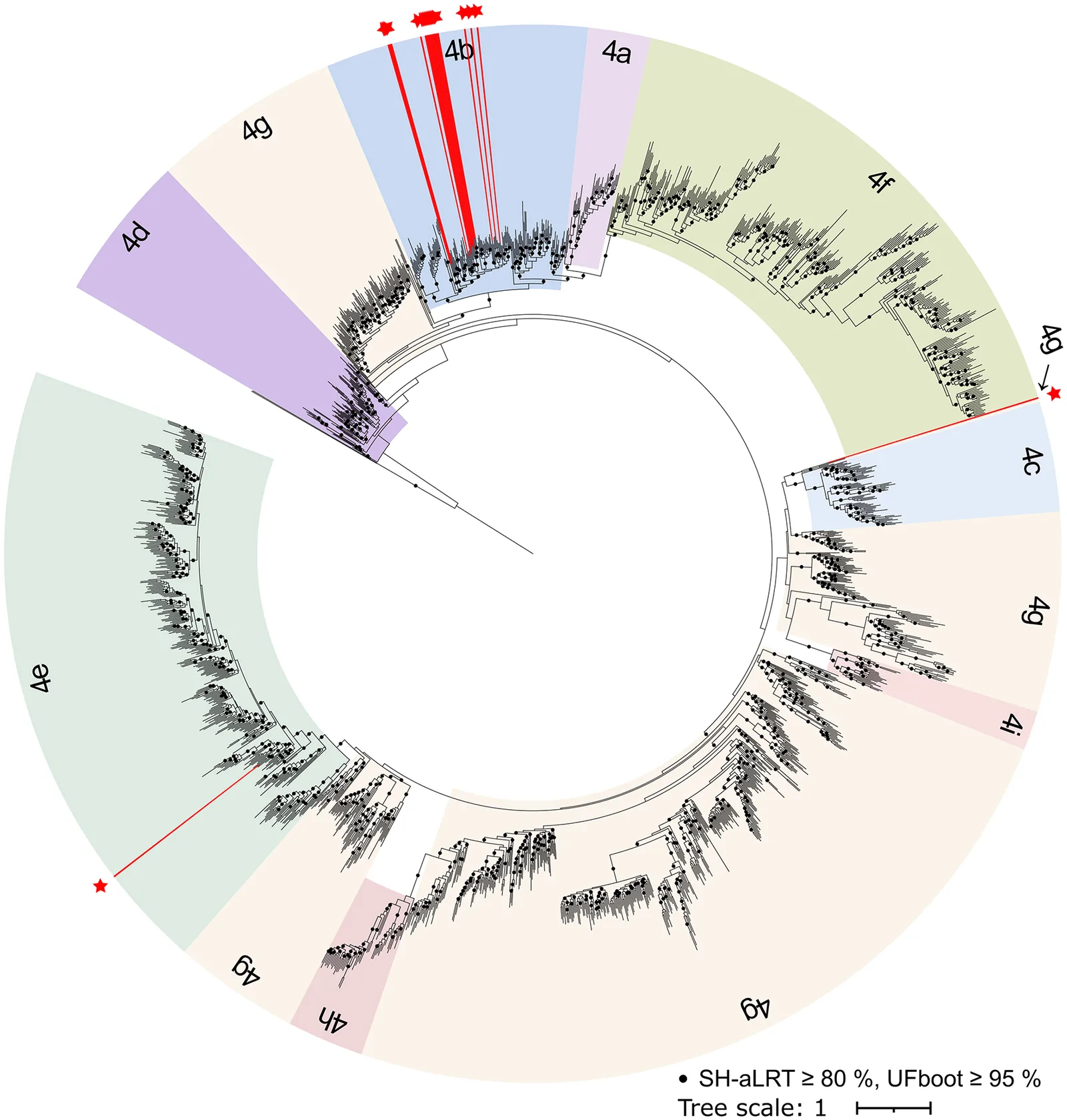
Maximum-likelihood phylogenetic tree of catalytic large subunits of Group 4 [NiFe/NiFeSe] hydrogenases. Representatives of Group 1 and Group 3 [NiFeSe] hydrogenases were included as outgroups. Black circles indicate branches supported by SH-aLRT ≥ 80 and ultrafast bootstrap ≥ 95 (1,000 replicates). Subgroups based on HydDB classification are highlighted with different background colors. Leaves containing Sec variants are highlighted with red lines and stars.

To assess the phylogenetic diversity of Sec variants, we constructed a maximum-likelihood tree of the catalytic large subunits using 2,022 representative sequences clustered at 80% sequence identity (Figure 2). Groups 4a, 4c, 4e, 4f, 4h, and 4i formed monophyletic clades, whereas Groups 4b and 4d were paraphyletic. Group 4g, previously described as a loosely clustered group (Søndergaard et al., 2016), was distributed across multiple distinct clades, indicating a polyphyletic pattern (Figure 2). The Sec variants were sporadically distributed in three subgroups—Group 4b (putatively formate-respiring), Group 4e (Ech-type, putatively ferredoxin-coupled), and Group 4g (putatively ferredoxin-coupled; Søndergaard et al., 2016). These results suggest that Sec variants independently emerged from Cys-type ancestors in multiple subclades, irrespective of their phylogeny and functional classification.

**Figure 3:**
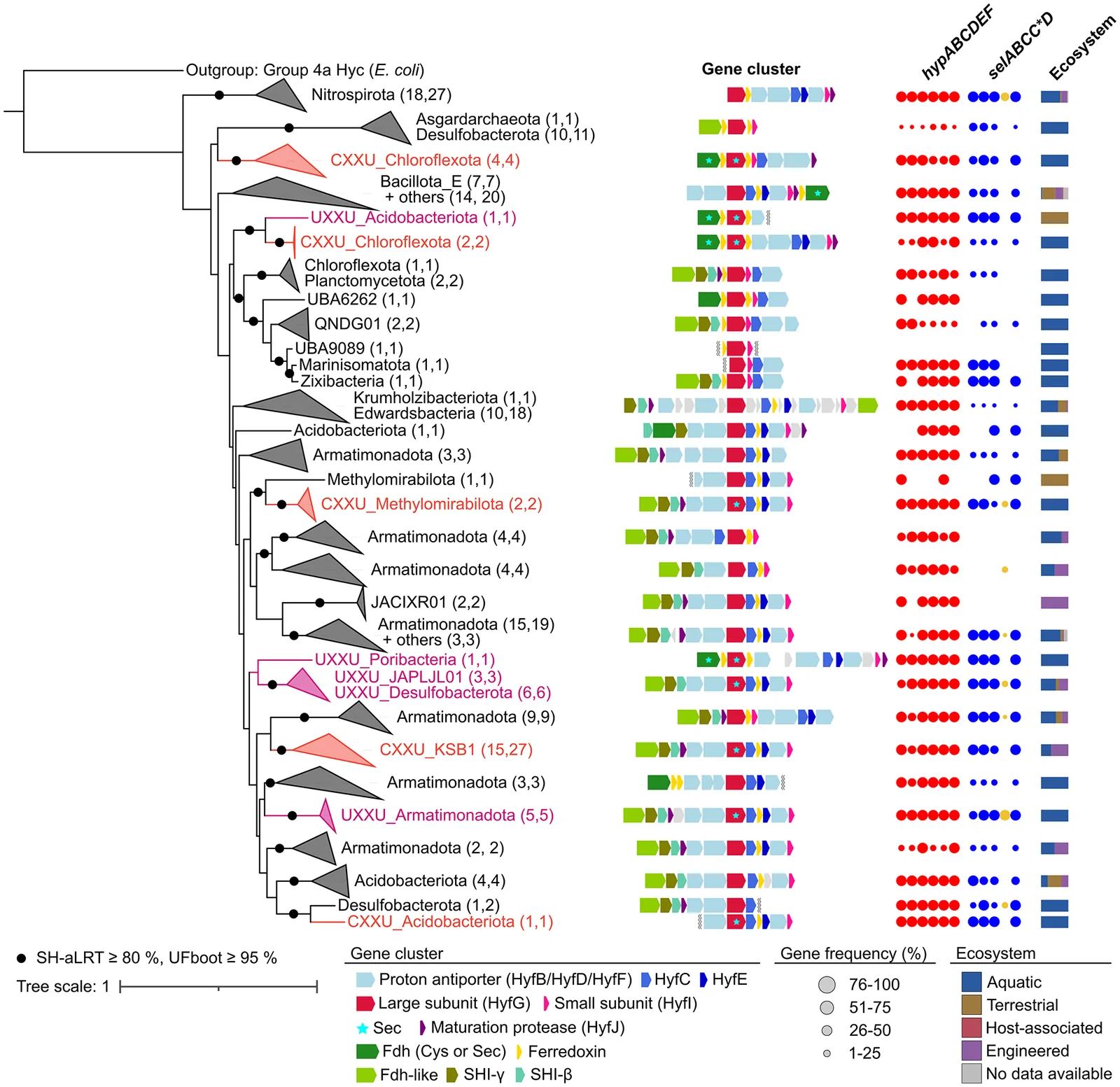
Maximum-likelihood phylogenetic tree of catalytic large subunits of Group 4b [NiFeSe] hydrogenases. The Group 4a Hyc hydrogenase from *Escherichia coli* was included as an outgroup. Black circles indicate branches supported by SH-aLRT ≥ 80 and ultrafast bootstrap ≥ 95 (1,000 replicates). Branches were grouped based on the type of the N-terminal Ni-binding motif, phylum, and gene cluster features. Branches and labels are colored according to motif type, with CXXC-, CXXU-, and UXXU-type sequences shown in black, red, and purple, respectively. Numbers in parentheses indicate the number of non-redundant sequences and the number of corresponding genomes. Representative gene clusters with the most complete annotations are shown. Proportions of genomes encoding *hypABCDEF* (red circles), *selABCD* (blue circles), and selC* (yellow circles) are indicated by circle size. Ecosystem distributions are shown as banded colors representing the proportions of metagenome-assembled genomes within each subclade, categorized into aquatic, terrestrial, engineered, and host-associated environments.

**Figure 4:**
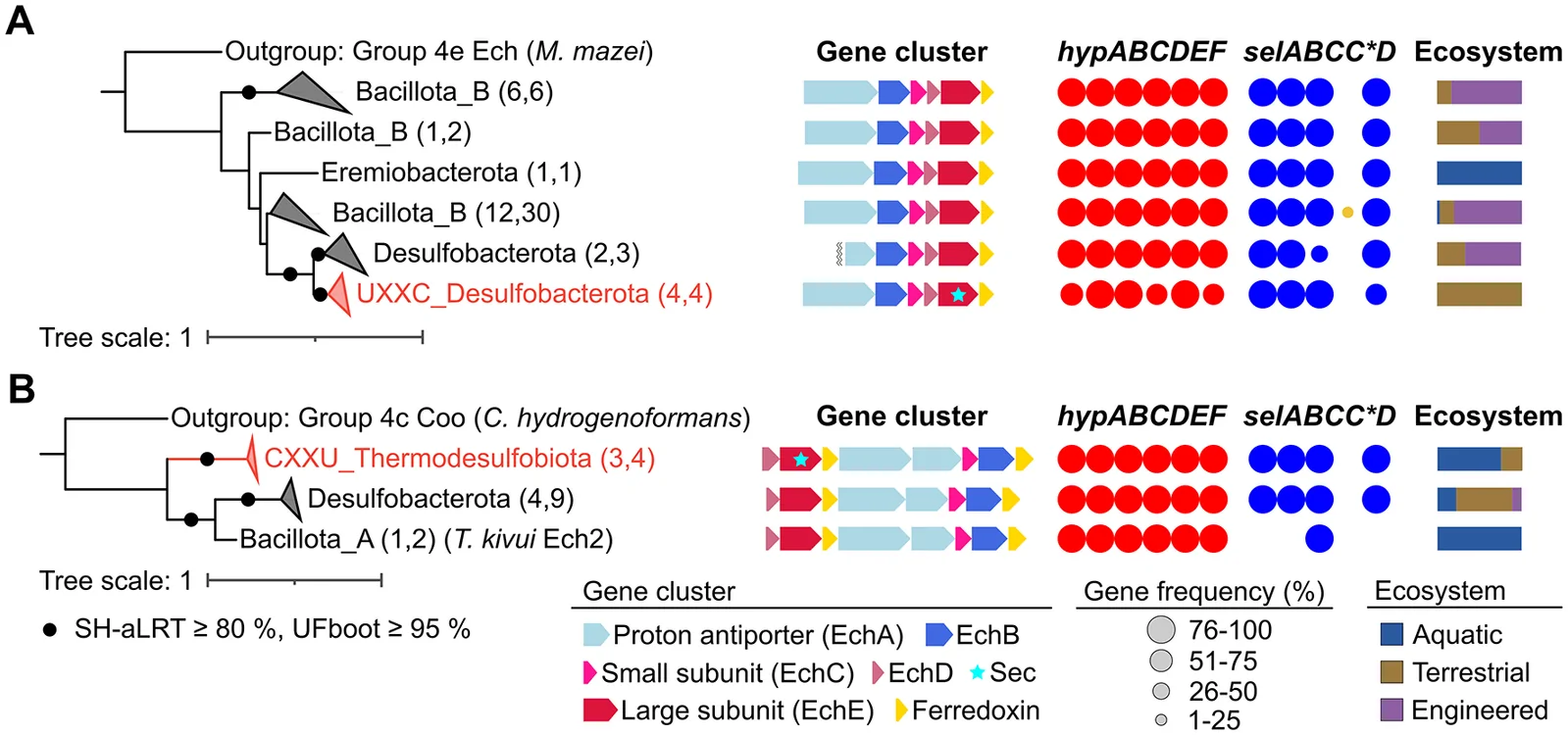
Maximum-likelihood phylogenetic trees of Groups 4e and 4g [NiFeSe] hydrogenases. **(A)** A tree of Group 4e catalytic large subunits. The Group 4e Ech hydrogenase from *Methanosarcina mazei* was included as an outgroup. **(B)** A tree of Group 4g catalytic large subunits. The Group 4c Coo hydrogenase from *Carboxydothermus hydrogenoformans* was included as an outgroup. Black circles indicate branches supported by SH-aLRT ≥ 80 and ultrafast bootstrap ≥ 95 (1,000 replicates). Branches were grouped based on the type of the N-terminal Ni-binding motif, phylum, and gene cluster features. Branches and labels corresponding to CXXU- and UXXC-type sequences are colored red. Numbers in parentheses indicate the number of non-redundant sequences and the number of corresponding genomes. Representative gene clusters with the most complete annotations are shown. Proportions of genomes encoding *hypABCDEF* (red circles), *selABCD* (blue circles), and selC* (yellow circles) are indicated by circle size. Ecosystem distributions are shown as banded colors representing the proportions of metagenome-assembled genomes within each subclade, categorized into aquatic, terrestrial, and engineered environments.

To further resolve phylogenetic and evolutionary relationships, we constructed additional maximum-likelihood trees focusing on subclades containing Sec-type sequences within Group 4b (Figure 3), Group 4e (Figure 4A), and Group 4g (Figure 4B). Frequent horizontal gene transfer (HGT) events at the phylum level were observed (Figures 3, 4). The majority of Sec-type subclades were associated with Cys-type sister groups from different phyla, suggesting that HGT at the phylum level co-occurred with the emergence of the Sec-type variants. Conversely, some Sec-type subclades possess Cys-type sister groups from the same phylum: Armatimonadota and Methylomirabilota in Group 4b, and Desulfobacterota in Group 4e. Notably, in Group 4e, both Sec-type (HYA15459.1) and Cys-type (HYA13691.1) sequences (~81% amino acid sequence identity) were present within a single Desulfobacterota genome (GCA_035625045.1), suggesting that the Sec-type variant may also have arisen via a gene duplication event.

A mosaic distribution of Sec-type and Cys-type subclades was observed within a single large subclade of Group 4b, with five and three Sec-type subclades harboring the CXXU and UXXU motifs, respectively, distributed across the tree (Figure 3). The UXXU type implies two independent Sec acquisition events; however, this phylogeny provides no clear evidence for a general stepwise emergence via the CXXU type. Only one exception may support such a transition: the UXXU type from Acidobacteriota and the CXXU type from Chloroflexota form a sister-group relationship, possibly reflecting an intermediate stage via the CXXU type.

Meanwhile, a UXXC-type subclade (Desulfobacterota) emerged in Group 4e, whereas a CXXU-type subclade (Thermodesulfobiota) emerged in Group 4g (Figure 4). These results indicate group-specific preferences for the position of Sec occurrence within the N-terminal CXXC motif.

### Structural and functional features of Group 4 [NiFeSe] hydrogenases

Genes encoding [NiFe] hydrogenase subunits are generally clustered in genomes (Greening et al., 2016; Søndergaard et al., 2016). We therefore analyzed genomic contexts to infer the subunit composition and function of Group 4 [NiFeSe] hydrogenases.

In Group 4b, a typical gene cluster encodes a catalytic large subunit, a catalytic small subunit, a HyfE-like subunit, a HyfC-like subunit, multiple proton antiporter subunits, multiple ferredoxin subunits, and an Fdh subunit, suggesting the formation of a formate hydrogenlyase-like complex (Figure 3). AlphaFold structural predictions of representative sequences further supported the assembly of these proteins into such complexes (Supplementary Figure 1).

Notably, approximately half of these Fdh sequences lacked the conserved Cys/Sec residues required for molybdenum coordination and catalysis (Figure 3 and Supplementary Figure 1B; Hartmann et al., 2016). The corresponding gene clusters co-occurred with genes encoding homologs of SHI-γ and SHI-β from the cytoplasmic hydrogenase I of *Pyrococcus furiosus*, which are involved in NADPH oxidation (Xiao et al., 2025). Based on AlphaFold structural predictions, the iron–sulfur cluster-mediated electron transfer network branches into three pathways that terminate at the catalytic large subunit, the SHI-γ subunit and the Fdh-like protein. This architecture suggests a role in electron bifurcation/confurcation coupling proton/H_2_, NADP^+^/NADPH, and an as-yet-unidentified substrate (Supplementary Figure 1B).

The gene clusters encoding Group 4e [NiFeSe] hydrogenases consist of a minimal gene set encoding a six-subunit complex, similar to those of Ech, which catalyzes ferredoxin-coupled energy conservation (Figure 4 and Supplementary Figure 1C). In contrast, Group 4g gene clusters share an organization identical to that of Ech2 from the thermophilic bacterium *Thermoanaerobacter kivui*, which is involved in carbon monoxide-dependent, ferredoxin-coupled energy conservation (Figure 4 and Supplementary Figure 1D; Baum et al., 2024).

In all clades, the genomic contexts of Sec-type sequences were similar to those of their closely related Cys-type counterparts, suggesting that the emergence of Sec does not involve drastic changes in complex composition.

### Hydrogenase maturation and Sec biosynthesis genes

Because Sec-type variants require both [NiFe] hydrogenase maturation and Sec utilization pathways, we investigated whether genes involved in hydrogenase maturation (*hypABCDEF*) and Sec biosynthesis and insertion (*selABCD*) were encoded in genomes harboring Sec-type and Cys-type hydrogenases.

**Figure 5:**
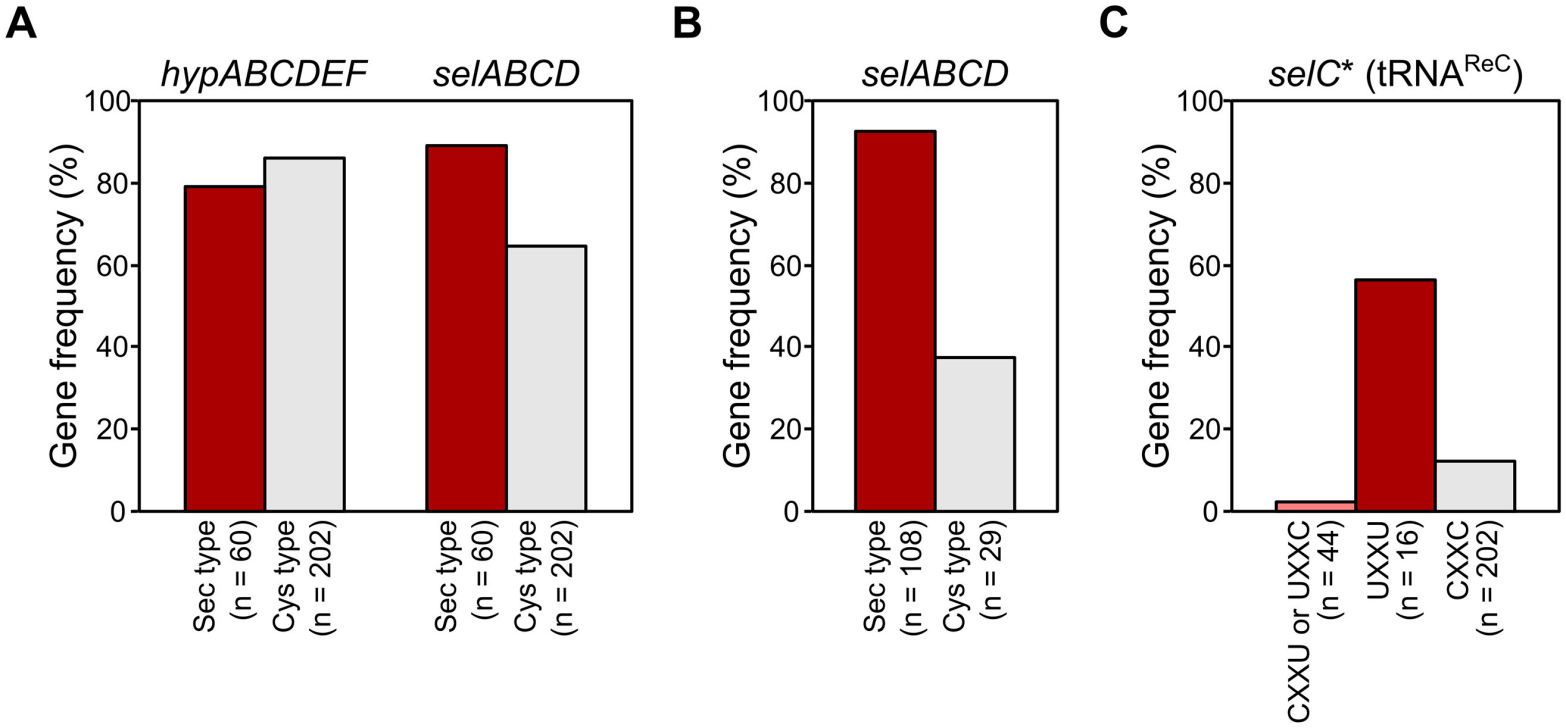
Frequencies of Sec-associated genes in genomes encoding Group 4 [NiFe/NiFeSe] hydrogenases. **(A)** Comparison of the frequencies of gene sets involved in hydrogenase maturation (*hypABCDEF*) and Sec biosynthesis (*selABCD*) between Sec- and Cys-type genomes. Frequencies were calculated while allowing for the absence of one gene within each gene set. **(B)** Frequencies of *selABCD* in genomes encoding either Sec- or Cys-type formate dehydrogenases, allowing for the absence of one gene. **(C)** Frequencies of selC* (tRNA^ReC^) in UXXU-type, CXXU- or UXXC-type, and CXXC-type genomes. Gene counts were calculated using the genomes shown in the phylogenetic trees in Figures 3 and 4.

In genomes harboring Sec variants, these gene sets were frequently incomplete, likely due to fragmentation in metagenomic assemblies (Figures 3, 4). When allowing one gene deletion from the four-gene set, the detection frequency of *selABCD* genes was significantly higher in Sec-type systems (~90%) than in Cys-type systems (~65%; *p* = 9.0 × 10^−5^ by Fisher's exact test; Figure 5A and Supplementary Figure 2A). In contrast, when allowing one gene deletion from the six-gene set, the detection frequency of *hypABCDEF* genes was comparable between Sec-type (~80%) and Cys-type systems (~86%). Although this pattern may partly reflect incomplete detection or the absence of Sec biosynthesis genes in some Cys-type genomes, the enrichment of *selABCD* in Sec-type genomes is consistent with a functional requirement for Sec utilization pathways. Additional putative selenoproteins (amino acid sequences containing U in the database) were identified in ~87% (52/60) of Sec-type genomes and ~68% (138/202) of Cys-type genomes (Supplementary Table 1), providing a potential explanation for the retention of Sec-utilization genes in many Cys-type genomes.

Notably, when comparing genomes encoding Sec-type Fdh with those encoding Cys-type Fdh on the phylogenetic tree in Figure 3, a strong enrichment of *selABCD* was observed in Sec-type genomes (93% vs. 38%; *p* = 1.8 × 10^−9^ by Fisher's exact test; Figure 5B and Supplementary Figure 2B). This suggests that the presence of Sec-type Fdh may impose stronger selective pressure to maintain Sec utilization pathways than Group 4 [NiFeSe] hydrogenases.

We further searched for tRNAs similar to the recently identified SelC^*^ (tRNA^ReC^), which enables decoding of UGA codons as Cys in the presence of downstream SECIS elements (Vargas-Rodriguez et al., 2018). Among UXXU-type Group 4 [NiFeSe] hydrogenase sequences, tRNA^ReC^-like tRNAs were found in 5/5 Armatimonadota, 2/6 Desulfobacterota, and 2/3 JAPLJL01 genomes in addition to canonical SelC (tRNA^Sec^; Figures 3, 5C; Supplementary Table 1). tRNA^ReC^ was significantly more frequent in genomes harboring the UXXU motif (~56%) than in other Sec-type genomes (~2.3%; *p* = 6.8 × 10^−6^ by Fisher's exact test), whereas it was detected at comparable frequencies in Sec-type (~17%) and Cys-type genomes (~13%; Figure 5C). Among the identified tRNA^ReC^-like tRNAs in UXXU-containing genomes, three carried a UCA anticodon and six carried a GCA anticodon (Supplementary Table 1). Both anticodon types have been reported to mediate UGA recoding in the presence of downstream SECIS elements (Vargas-Rodriguez et al., 2018).

### Structural features of SECIS elements

**Figure 6:**
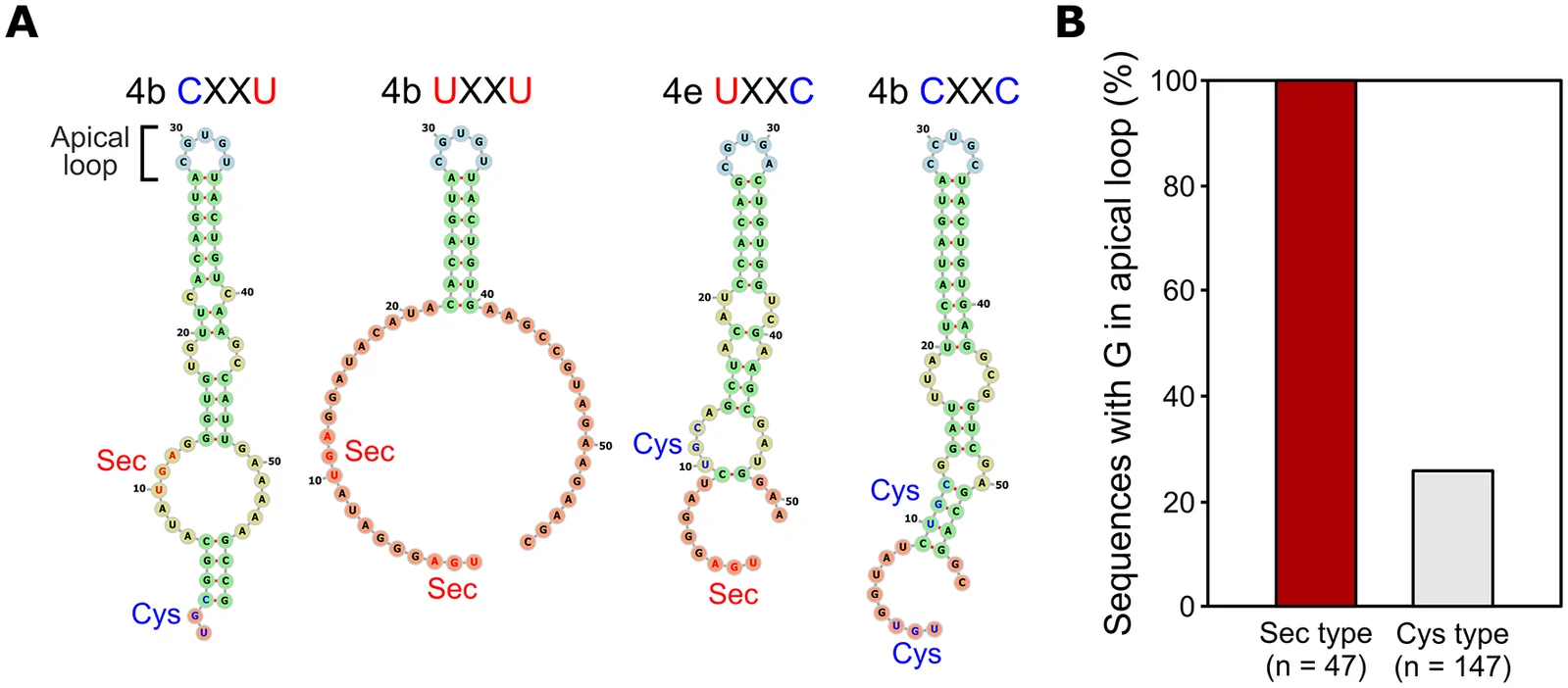
Structural features of SECIS elements. **(A)** Predicted secondary structures of representative SECIS elements from Group 4b CXXU-type (JW963_00885, Chloroflexota), Group 4b UXXU-type (NT056_09300, JAPLJL01), Group 4e UXXC-type (VEF33_14075, Desulfobacterota), and Group 4b CXXC-type (JSV82_09380, Chloroflexota) sequences. **(B)** Conservation of guanine at either the first or second position in the apical loop.

In Sec-type representatives from each subclade, mRNA segments spanning the UGA codon and its ~50 nt downstream region were predicted to form SECIS-like stem–loop structures (Figure 6 and Supplementary Figure 3). Most of these exhibited a typical architecture consisting of an apical loop, upper stem, internal loop, and lower stem, whereas some were predicted to lack the internal loop and lower stem.

In addition, either the first or second position of the apical loop was completely occupied by guanine (100%), consistent with the consensus sequence of bacterial SECIS elements (Zhang et al., 2026). In contrast, closely related Cys-type representatives were also predicted to form SECIS-like structures downstream of the UGC/U codon; however, guanine conservation in the apical loop was substantially lower (~26%). Accordingly, guanine conservation was significantly enriched in Sec-type sequences (*p* = 8.9 × 10^−22^ by Fisher's exact test).

To examine the evolutionary consequences of mutations affecting conserved guanines within SECIS elements, we analyzed the corresponding coding sequences in each group (Supplementary Figure 4). In Group 4b, the conserved guanine at the second position of the apical loop corresponded to the third position of a threonine codon (ACG). Reduced conservation of this guanine in Cys-type sequences was associated with either synonymous substitutions or amino acid replacements to Cys, valine, serine, or alanine. In Group 4e, the conserved guanine corresponded to the first position of a valine codon (GUG), and its loss in Cys-type sequences was predominantly associated with valine-to-isoleucine/methionine/alanine/Cys substitutions. In Group 4g, conserved guanines corresponded to the third position of a tryptophan codon (UGG) and the first position of a valine codon (GUC). Whereas the latter remained highly conserved in Cys-type sequences, loss of the former was associated with tryptophan-to-phenylalanine substitutions. These substitutions generally involved amino acids with similar physicochemical properties.

### Environmental distribution of Group 4 [NiFeSe] hydrogenases

**Figure 7:**
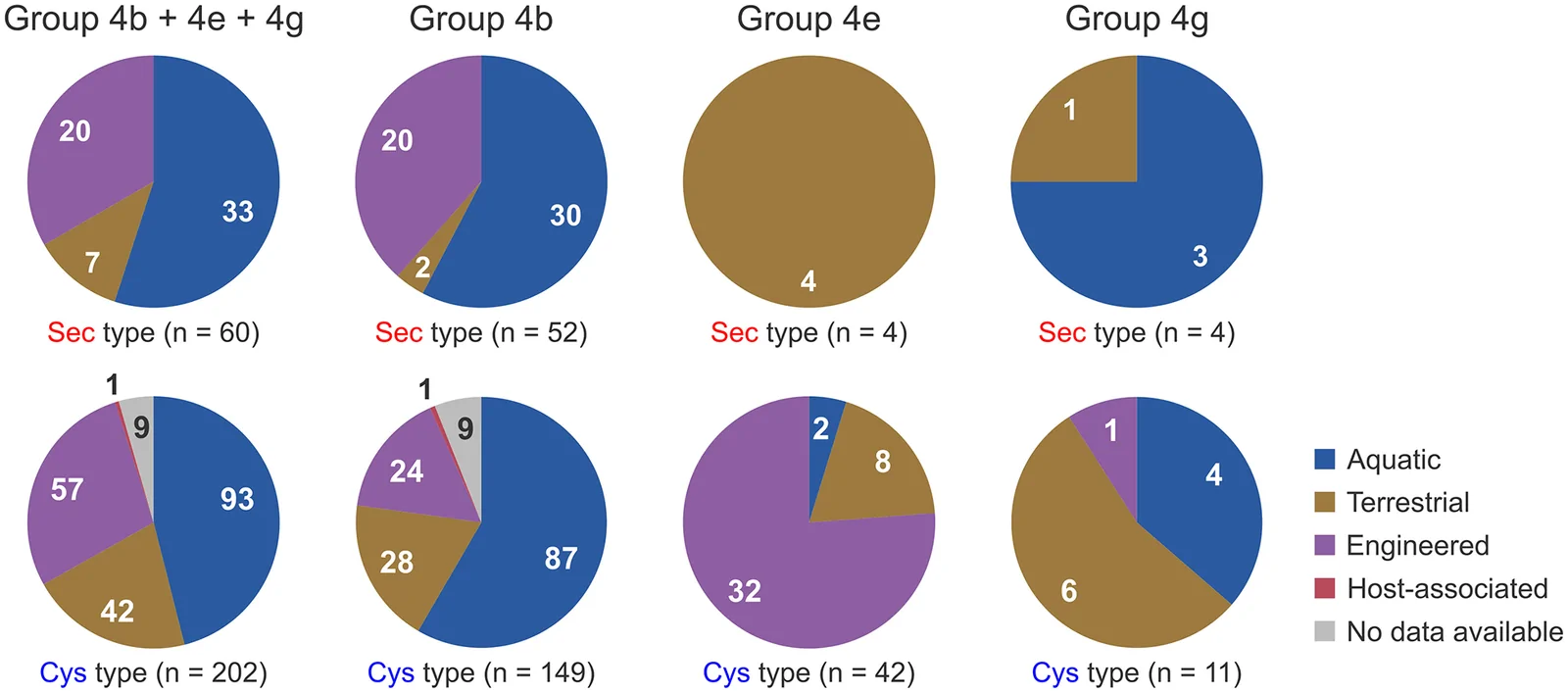
Distribution of ecosystem types among genomes encoding Groups 4b, 4e, and 4g [NiFe/NiFeSe] hydrogenases. Pie charts show the proportions of ecosystem types for each subgroup, comparing Sec-type and Cys-type genomes. Ecosystem information associated with metagenome-assembled genomes was categorized into aquatic, terrestrial, engineered, and host-associated environments.

To explore the ecological contexts associated with Group 4 [NiFeSe] hydrogenases, we analyzed ecosystem metadata linked to metagenome-assembled genomes harboring these sequences. Overall, Sec-type sequences were largely distributed in aquatic environments (55%), including wastewater, hot springs, and freshwater systems (Figures 3, 4, 7; Supplementary Table 1). This was followed by engineered environments (33%) and terrestrial environments (12%). A similar distribution pattern was observed for closely related Cys-type sequences, with a relatively higher proportion in terrestrial environments (20%) compared to Sec-type sequences.

When examined at the subgroup level, the differences in distribution between Sec-type and Cys-type sequences were more pronounced (Figure 7 and Supplementary Table 1). In Group 4b, Sec-type sequences were enriched in engineered environments and less common in terrestrial environments compared to closely related Cys-type sequences. In contrast, in Group 4e, all Sec-type sequences were derived from terrestrial environments (soil-derived metagenomes from wetlands and tundra environments), whereas Cys-type sequences were more frequently associated with engineered environments. In Group 4g, no clear distribution pattern was observed for Cys-type sequences, whereas Sec-type sequences were biased toward aquatic environments.

Notably, three Sec-type sequences in Group 4g were identified in isolate genomes belonging to *Thermodesulfobium* spp. (*T. narugense, T. acidiphilum*, and *T. fumaratoxidans*), obligately anaerobic, thermophilic sulfate-reducing bacteria that have been isolated from hot spring sediments and geothermal soils (Figure 4B and Supplementary Table 1; Mori et al., 2003; Frolov et al., 2017; Maltseva et al., 2026). Given that Cys-type counterparts in Desulfobacterota are also thermophilic (*Thermodesulfobacterium* spp.), these findings suggest a scenario in which HGT under anaerobic conditions in geothermal environments was followed by the emergence of Sec-type enzymes.

## Discussion

This study demonstrates that the emergence of Sec-type variants in Group 4 [NiFe] hydrogenases, which has previously been overlooked, represents a rare event (~0.2%) but has occurred independently across multiple bacterial phyla and subgroups of Group 4 enzymes (Figures 1, 2). The repeated emergence of Sec-type variants without altering gene cluster organization, consistent with observations in Groups 1 and 3, suggests that Sec primarily fine-tunes enzymatic properties rather than restructuring the overall complex (Garcin et al., 1999; Baltazar et al., 2011; Marques et al., 2017).

A striking feature of Group 4 [NiFeSe] hydrogenases is the exclusive localization of Sec residues within the N-terminal Ni-binding motif of the catalytic large subunit (Figure 1), in contrast to the C-terminal localization observed in Groups 1 and 3 (Garcin et al., 1999; Baltazar et al., 2011; Marques et al., 2017). Exceptions have been reported in which two sequences from Group 1 and one from Group 3 possess an N-terminal CXXU motif, where Sec is predicted to bridge Ni and Fe atoms (Baltazar et al., 2011). However, the UXXC and UXXU motifs identified here represent previously unrecognized variants, where the first Sec likely acts as a non-bridging ligand coordinating only the Ni atom. Engineered substitution of each of the four Cys residues in a Group 1 [NiFe] hydrogenase with Sec has been shown to retain catalytic activity, although the catalytic properties were altered (Evans et al., 2021, 2024). These findings suggest that Cys-to-Sec substitutions are not necessarily incompatible with hydrogenase function. Notably, the Sec in the C-terminal UXXC motif has been linked to specific functional advantages, including oxygen tolerance and enhanced H_2_ production (Parkin et al., 2008; Marques et al., 2017; Evans et al., 2021, 2024). It is therefore intriguing that Sec incorporation in Group 4 is restricted to the N-terminal motif. Understanding the structural and functional basis of this positional constraint will require further experimental investigation.

Phylogenetic analyses revealed that Sec-type variants in Group 4 have emerged independently from Cys-type ancestors at least three times (Figure 2). However, it should be noted that the mosaic distribution of Sec-type and Cys-type sequences within a single subclade of Group 4b allows two alternative interpretations: repeated independent gain of Sec, or secondary loss from Sec-type ancestors (Figure 3). The SECIS-like stem–loop structures observed in closely related Cys-type sequences suggest a pre-existing capacity to function as SECIS elements, potentially facilitating the emergence of Sec-type variants. Alternatively, these structures may represent remnants of functional Sec incorporation systems, with mutations in the conserved guanine of the apical loop occurring after reversion to the Cys type. Disruption of SECIS secondary structure accompanying mutation to the Cys type has recently been reported for SelD (Inoue et al., 2024), supporting the latter possibility. Notably, loss of guanine conservation in Cys-type sequences is typically associated with either synonymous substitutions or conservative amino acid replacements (Supplementary Figure 4), suggesting that loss of SECIS-associated sequence constraints can occur with only minor effects on the encoded protein.

The gain or loss of Sec appeared to be associated with phylum-level HGT events (Figures 3, 4). However, HGT occurs frequently across the evolutionary landscape of Group 4 [NiFe] hydrogenases (Greening et al., 2016), precluding definitive conclusions. Nevertheless, it is plausible that differences in gene sets (e.g., those involved in Sec biosynthesis) between donor and recipient organisms during HGT events, as well as differences in environmental selective pressures, could drive the gain or loss of Sec (Zhang et al., 2006). Although subgroup-specific biases were observed in ecosystem distribution, HGT at the phylum level makes it difficult to determine whether environmental conditions themselves exert direct selective pressure.

One notable feature of Group 4 [NiFeSe] hydrogenases is the presence of UXXU motifs containing two closely spaced putative Sec residues. Analysis of SelB homologs encoded in genomes harboring UXXU-type variants revealed no obvious deviations from canonical bacterial SelB proteins (Kromayer et al., 1999) and no additional domains potentially associated with decoding of SECIS elements (Supplementary Figure 5). In Group 4b, transitions between UXXU- and CXXU-type motifs are unlikely to require major alterations of SECIS structure because the relevant substitutions occur upstream of the predicted SECIS region, and the core SECIS architecture is highly conserved between the two motif types (Supplementary Figure 4A). These observations raise the possibility that both UGA codons in UXXU-type variants are decoded under the control of a single downstream SECIS element. Nevertheless, additional factors may be involved in the decoding of UXXU motifs.

In this context, it is noteworthy that genomes harboring UXXU motifs exhibited a significantly higher prevalence of tRNA^ReC^ (Figure 5C). Although the functional partitioning between the tRNA^Sec^ and tRNA^ReC^ systems remains unclear (Vargas-Rodriguez et al., 2018), the co-existence of both tRNAs may facilitate the evolutionary transition toward Sec-type enzymes through an additional mutation from UGC/U to UGA. It should also be noted that, in genomes encoding tRNA^ReC^, either one or both UGA codons may be translated as Cys rather than Sec, representing a limitation inherent to bioinformatics-based analyses.

## Data Availability

The original contributions presented in the study are included in the article/Supplementary material, further inquiries can be directed to the corresponding authors.
