# Supplementary material for: In silico discovery of selenocysteine-containing variants of Group 4 [NiFe] hydrogenases
Source: Front Microbiol. 2026 Sep 3;17:1925269. doi: 10.3389/fmicb.2026.1925269 (PMC13582414; doi:10.3389/fmicb.2026.1925269)
Supplement: Supplementary file 1 [file Data_Sheet_1.pdf]

Supplementary Material

In silico discovery of selenocysteine-containing variants of Group 4 [NiFe] hydrogenases

Masamitsu Takano, Masao Inoue*, Riku Aono, Anna Ochi, Hisaaki Mihara*

***Correspondence:** Masao Inoue, mainoue@fc.ritsumei.ac.jp; Hisaaki Mihara, mihara@fc.ritsumei.ac.jp

1 Supplementary Tables

Supplementary Table 1. Metadata for Group 4b, 4e, and 4g [NiFe/NiFeSe] hydrogenase subgroups related to Figures 3 and 4, including information on proteins, genomes, taxonomy, ecosystem, putative SECIS elements, [NiFe] hydrogenase maturation genes, and selenocysteine biosynthesis genes.
Presented as a separate MS Excel file.

2 Supplementary Figures

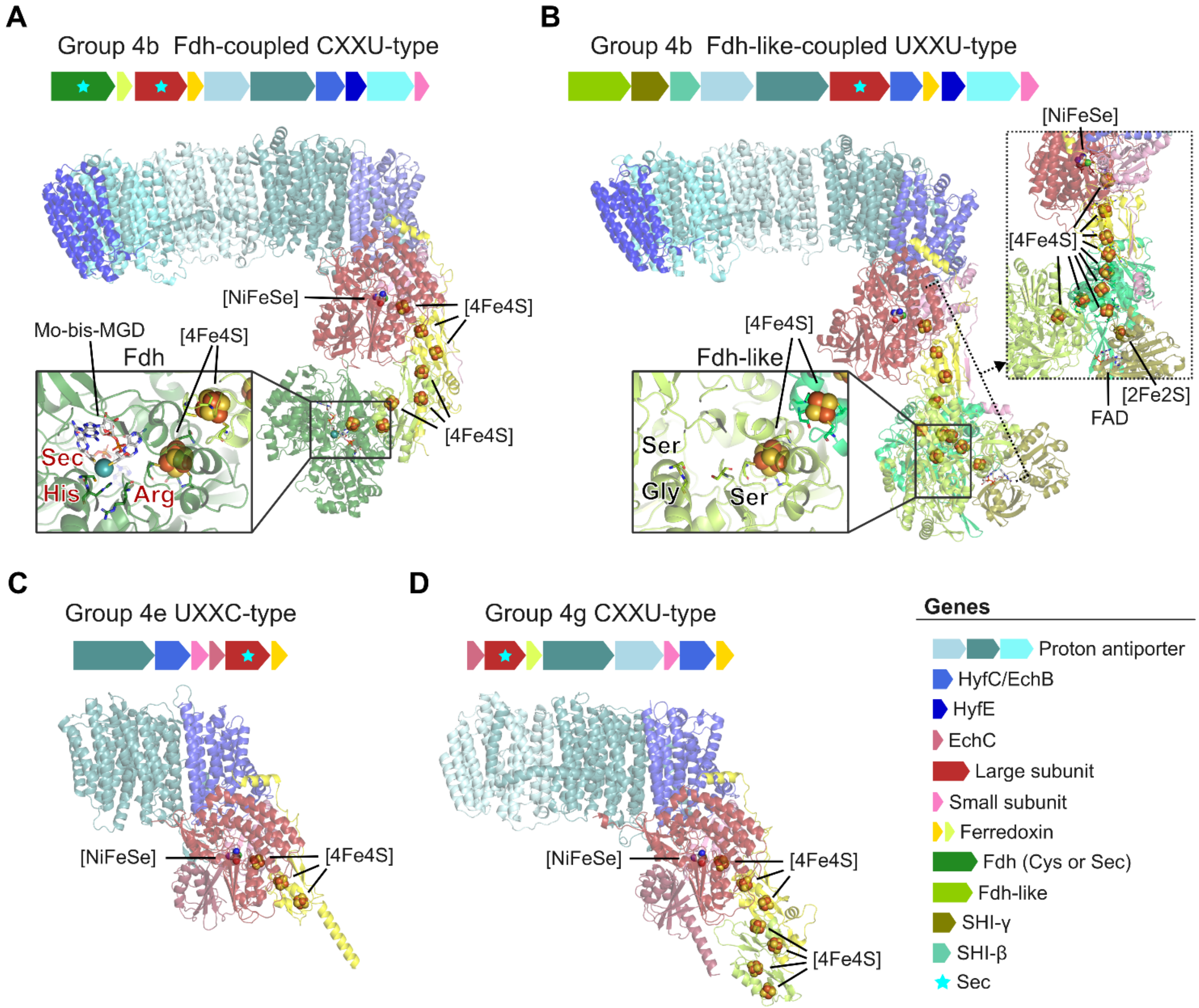

Supplementary Figure 1. Predicted structures of representative [NiFeSe] hydrogenase complexes. **(A)** Group 4b CXXU-type from Chloroflexota (MCX6010236.1–MCX6010246.1). **(B)** Group 4b UXXU-type from Desulfobacterota (MDI6776830.1–MDI6776841.1) **(C)** Group 4e UXXC-type from Desulfobacterota (HVO65047.1–HVO65053.1) **(D)** Group 4g CXXU-type from Thermodesulfobiota (WP_108308809.1–WP_108308817.1). For catalytic large subunits containing Sec, full-length sequences with UGA readthrough were used. To avoid program errors, Sec residues were substituted with Cys. C-terminal regions subject to post-translational processing were removed prior to analysis. Gene clusters are shown above the predicted structures, with colors matching the corresponding subunit models. Sec positions are indicated by cyan asterisks.

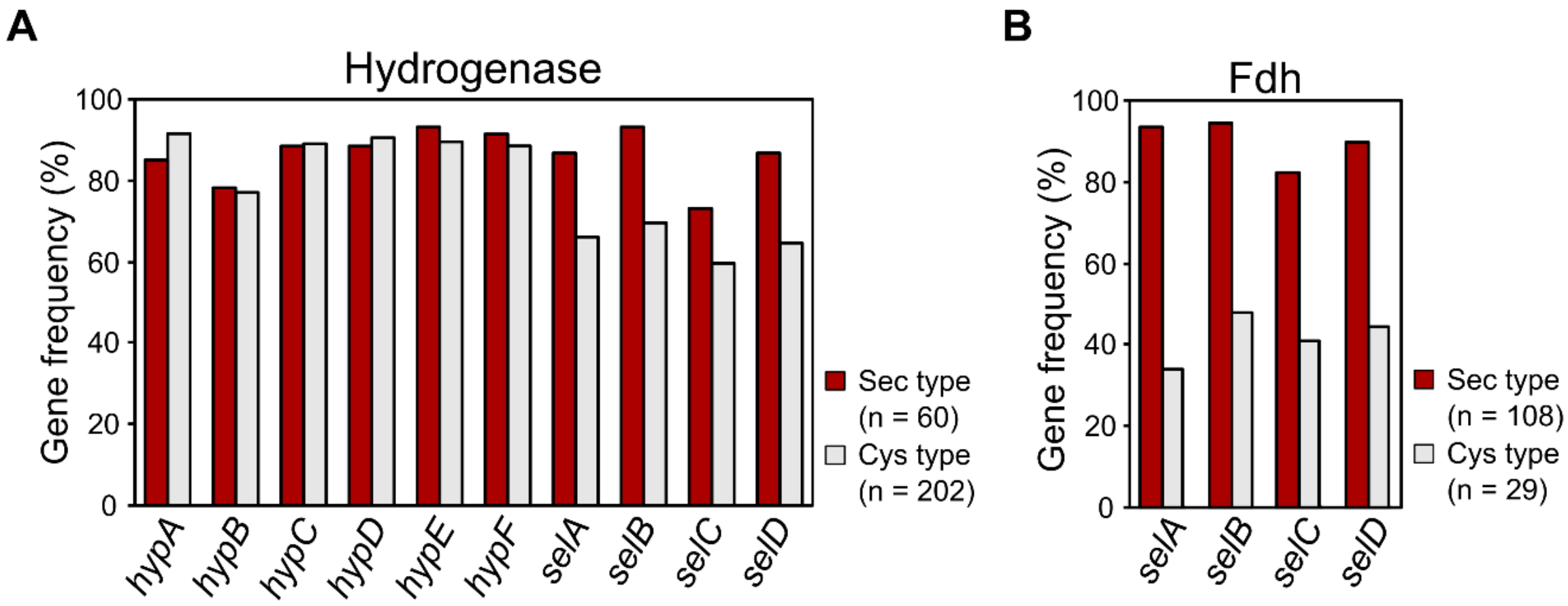

Supplementary Figure 2. Frequencies of Sec-associated genes in genomes encoding Group 4 [NiFe/NiFeSe] hydrogenases, related to Figure 5. **(A)** Comparison of the frequencies of genes involved in hydrogenase maturation (*hypABCDEF*) and Sec biosynthesis (*selABCD*) between Sec-type and Cys-type genomes. **(B)** Frequencies of *selABCD* in genomes encoding either Sec-type or Cys-type formate dehydrogenases. Gene counts were calculated using the genomes shown in the phylogenetic trees in Figs. 3 and 4.

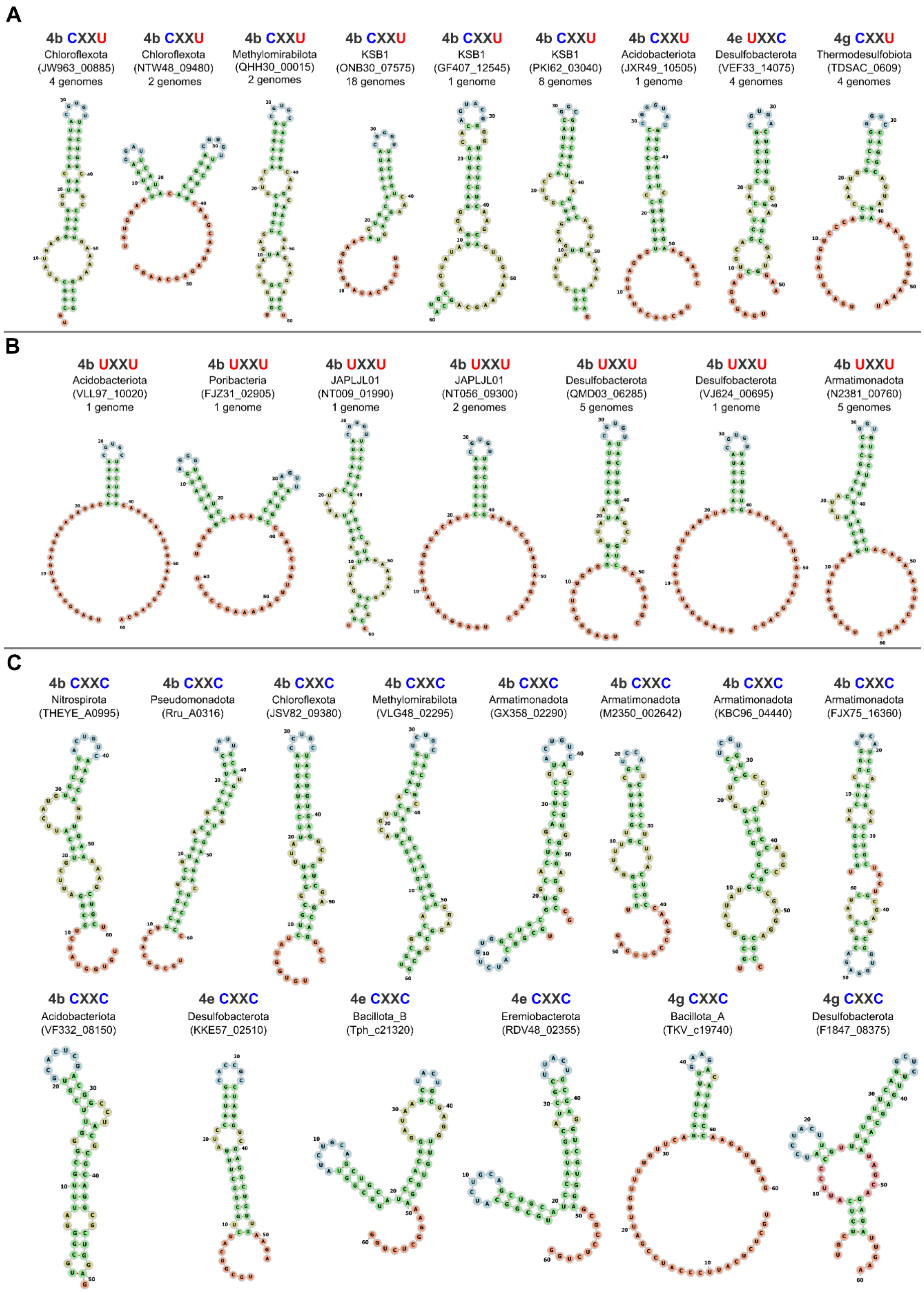

Supplementary Figure 3. Predicted secondary structures covering all major SECIS-like structural patterns identified among Sec- and Cys-type sequences. **(A)** CXXU-type or UXXC-type. **(B)** UXXU-type. **(C)** CXXC-type.

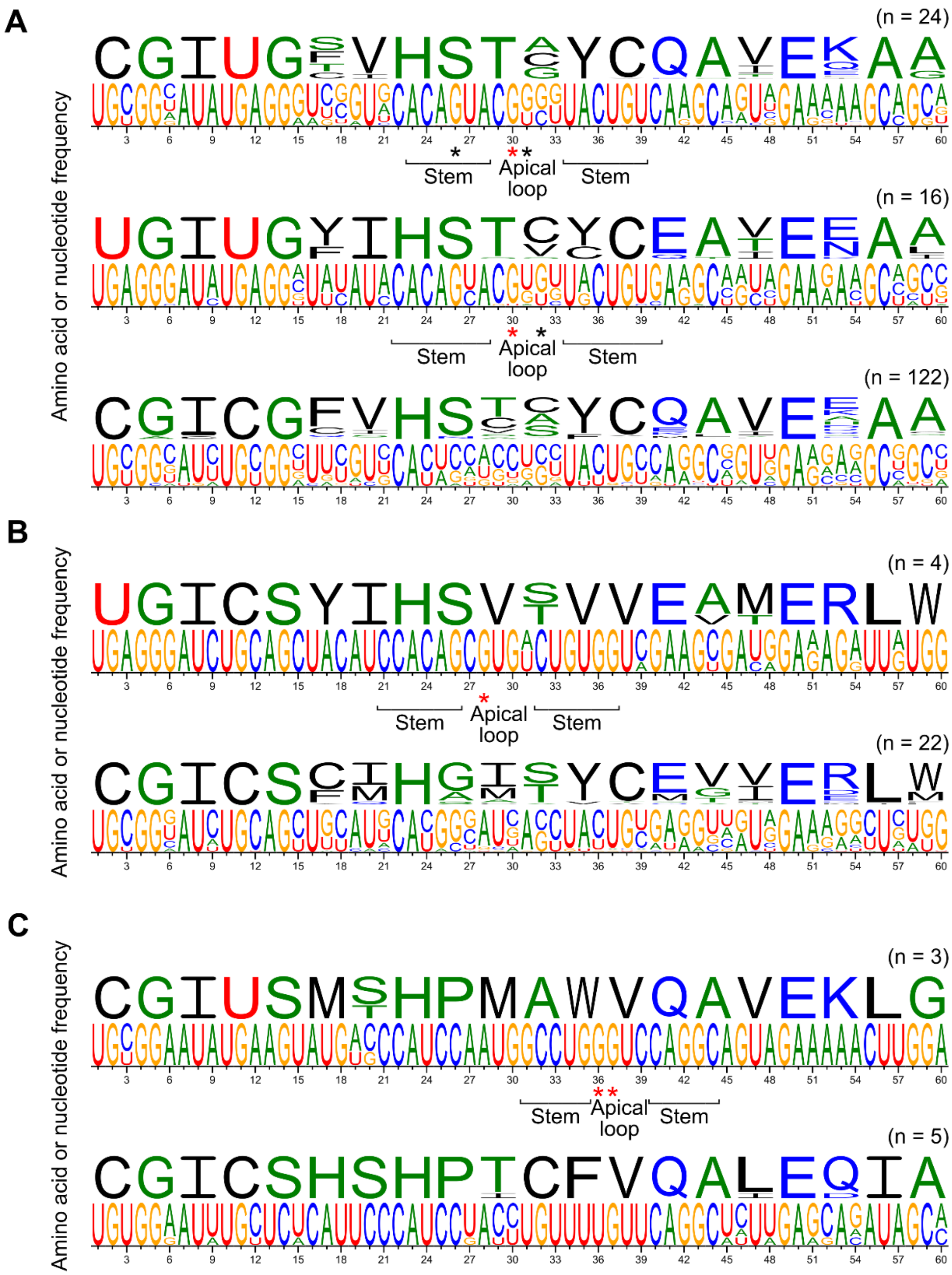

Supplementary Figure 4. Amino acid and nucleotide frequencies of Sec- and Cys-type sequences in each group. The conserved N-terminal CXXC motif and the following 16 amino acid residues (corresponding to 60 nucleotides) were visualized using WebLogo3 for Group 4b (A), Group 4e (B), and Group 4g (C). Amino acid residues were colored according to hydrophobicity, and the U residues were highlighted in red. For Sec-type sequences, representative core stem-loop structures are indicated. Red asterisks indicate the conserved apical-loop guanine found in most sequences, whereas black asterisks indicate guanine positions in alternative SECIS-like structures.

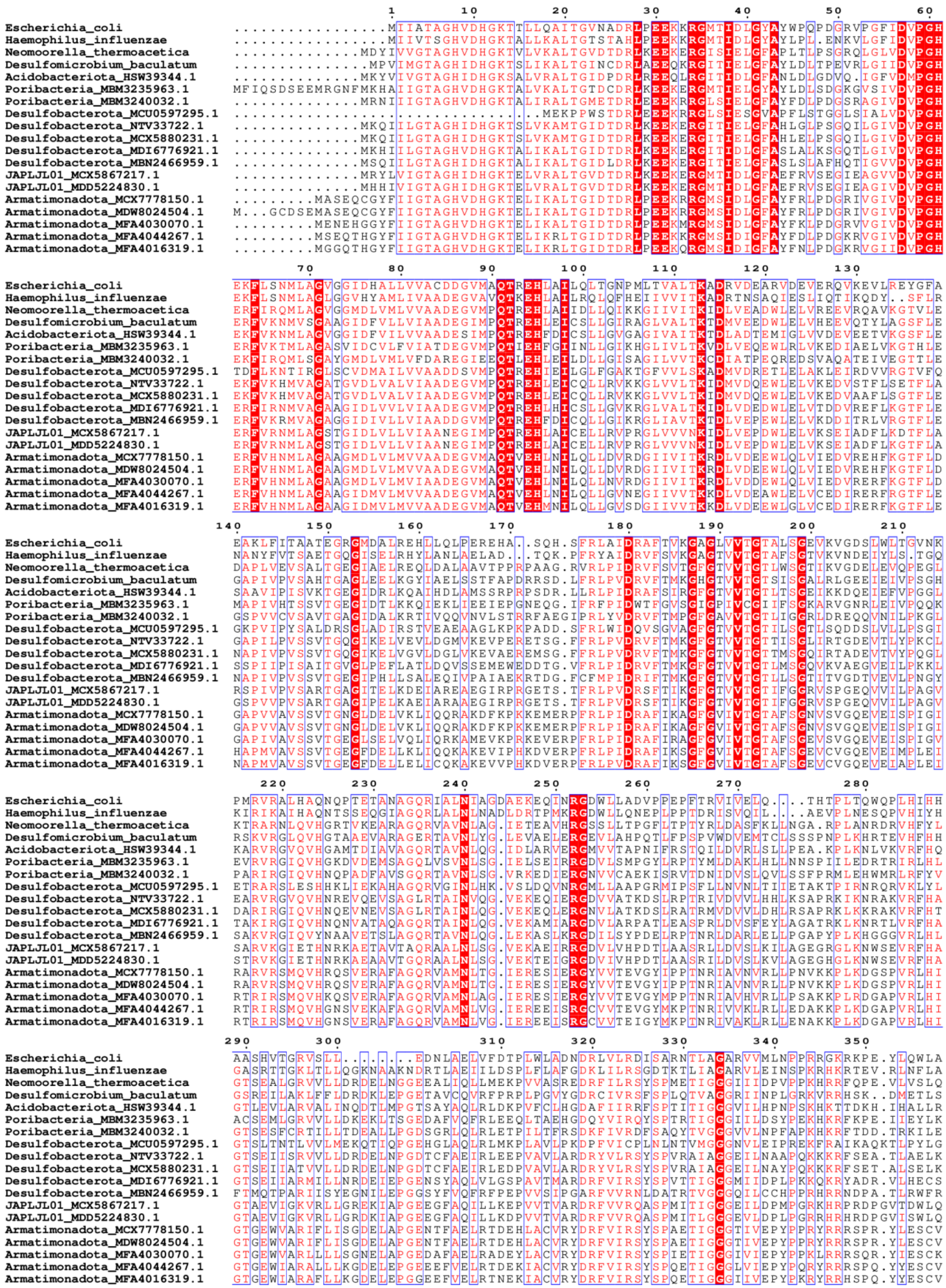
Escherichia_coli
Haemophilus_influenzae
Neomoorella_thermoacetica
Desulfomicrobium_baculatum
Acidobacteriota_HSW39344.1
Poribacteria_MBM3235963.1
Poribacteria_MBM3240032.1
Desulfobacterota_MCU0597295.1
Desulfobacterota_NTV33722.1
Desulfobacterota_MCX5880231.1
Desulfobacterota_MDI6776921.1
Desulfobacterota_MBN2466959.1
JAPLJL01_MCX5867217.1
JAPLJL01_MDD5224830.1
Armatimonadota_MCX7778150.1
Armatimonadota_MDW8024504.1
Armatimonadota_MFA4030070.1
Armatimonadota_MFA4044267.1
Armatimonadota_MFA4016319.1

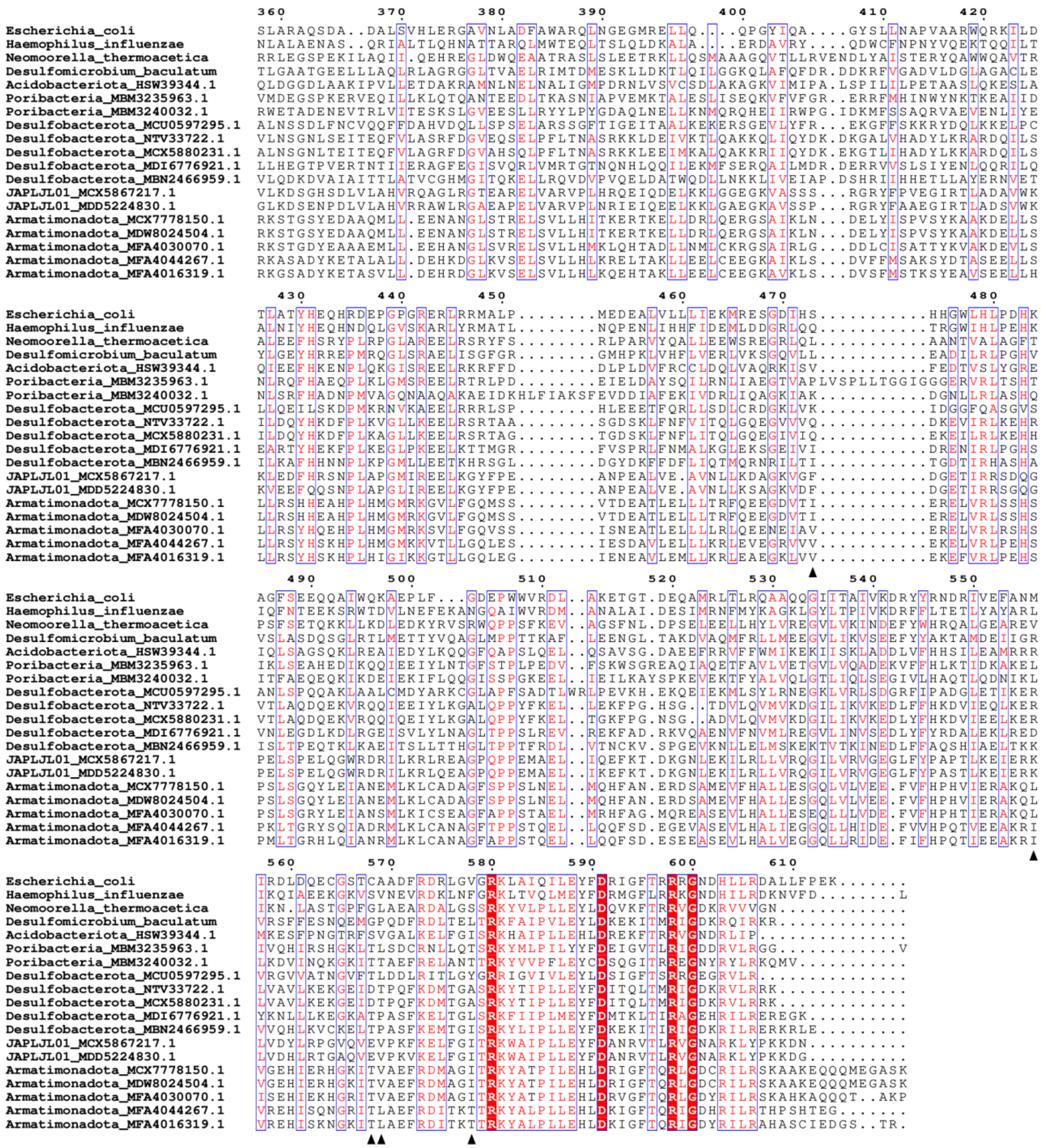

Supplementary Figure 5. Multiple sequence alignment of SelB proteins encoded in genomes harboring UXXU-type [NiFeSe] hydrogenases. Representative bacterial SelB proteins from *Escherichia coli* (WP_000582468.1), *Haemophilus influenzae* (AAC22366.1), N*eomoorella thermoacetica* (WP_011393980.1), and *Desulfomicrobium baculatum* (CAA68184.1) are included. Black triangles indicate residues important for recognition of the SECIS loop structure (Kromayer et al., 1999). The alignment was visualized using ESPript version 3.2.
